# Age and Education Effects on a Novel Syntactic Assessment Battery for Elderly Adults

**DOI:** 10.3389/fpsyg.2021.639866

**Published:** 2021-06-18

**Authors:** Jee Eun Sung, Heekyung Ahn, Sujin Choi, Kiseop Lee

**Affiliations:** ^1^Department of Communication Disorders, Ewha Womans University, Seoul, South Korea; ^2^Department of Statistics, Purdue University, West Lafayette, IN, United States

**Keywords:** syntactic assessment battery, sentence comprehension, age effect, education effect, screening linguistic tool

## Abstract

The purpose of this study was to delineate the properties of a novel syntactic assessment battery and to present descriptive data on normal elderly individuals. We administered the Syntactic Assessment Battery (hereinafter SAB) using a sentence-picture paradigm to 195 normal elderly adults in three age groups (60–69, 70–79, and 80–90) and five educational levels (No formal education, Elementary School Graduation, Middle School Graduation, High School Graduation, College Graduation and Above). A multiple linear regression model was applied to verify the age and education effects. A summary of results indicated that the SAB effectively detected age and education effects. People generally demonstrated worse performance as they aged but better performance as their educational levels increased. People with high school education and above generally demonstrated stronger performance on the test, although educational effects were not significantly different between elementary and middle school graduation groups. The current novel syntactic assessment battery can serve as a screening measure that sensitively detects age and education effects.

## Introduction

Although sentence comprehension abilities have attained increased attention in association with aging-related changes in cognitive and linguistic performance, evidence on aging-related decline in sentence processing abilities may vary depending on the task complexity and response modality. Some researchers argued that sentence processing abilities decline as people age (Burke and College, 1997; Waters and Caplan, 2001; Caplan et al., 2011), while others suggested that some other aspects of sentential anomality detection abilities are preserved (e.g., Tyler et al., 2010; Meunier et al., 2014).

Several versions of sentence comprehension tests are available for English speakers to detect aging effects. Assessment paradigms vary, from sentence-picture matching task (Caplan et al., 1997; Small et al., 1997) to syntactic plausibility judgment tests (Rochon et al., 1994; Meyer et al., 2012). Such tests often tap into syntactic parsing abilities which encompass an array of capabilities to identify the syntactic structures and interpret the meaning of a sentence by integrating syntax and semantics. In the process of interpreting a sentence, the interface between syntax and semantics comes into play. When the semantic top-down cues are salient in a sentence, listeners or readers are able to process and understand the sentence more easily. For example, if the two nouns “cat” and “mouse” are presented along with the verb “chase,” people are more likely to predict the relationship between the two nouns by constructing a sentence such as “the cat is chasing the mouse” rather than “the mouse is chasing the cat.” This is due to people’s heavy reliance on top-down semantic processing that dictates it is more semantically plausible for the cat to chase the mouse than the reverse. Considering the influence of semantic processing on syntactic parsing (who is doing the action vs. who is receiving the action), researchers have attempted to develop sentence comprehension tests by controlling the effects of top-down semantic properties on syntactic abilities. One possible method to control these effects is to employ semantically reversible sentences in which the two nouns in the sentence are equally plausible to do or receive the action, for example using nouns such as animals and humans (Kim et al., 2012).

A Previous study from Caramazza and Zurif (1976) reported that sentence comprehension abilities are heavily influenced when semantically reversible sentence types are used to assess aphasic individuals’ abilities to process sentences. This seminal report shed light on the importance of selecting the syntactic assessment paradigm, given that there are certain assessment paradigms, which sensitively detects impaired abilities to parse thematic roles of the noun phrases. Prior to that report, it had been believed that abilities to comprehend sentences are relatively preserved for people with Broca’s aphasia. However, the authors reported that individuals with Broca’s aphasia presented chance-level performance in semantically reversible sentences (e.g., The boy chases the girl) but performed significantly better than chance in non-reversible sentences (e.g., The boy eats an apple). The results suggested that semantic balance of the thematic roles from the noun phrases needs to be controlled when testing the syntactic abilities.

However, it is not easy to fully control the semantic features associated with nouns, for example, with animals and humans as subjects and objects in a sentence, given that so many factors need to be taken into consideration such as predatory relations for animals and relational hierarchy for humans. Some tests such as the Revised Token Test (McNeil and Prescott, 1978) minimized the issues associated with the semantic relations among the noun phrases in a sentence by having all sentences use shape-related nouns with color- and size-related adjectives in the token tests. By minimizing the top-down semantic bias on the relationship between the nouns in a sentence, the token test successfully tapped into syntactic abilities. Due to these features, previous studies using the Revised Token Test suggested that this paradigm (i.e., with rigorously controlled top-down lexical activation) is strongly related to working memory capacity by increasing task demands (e.g., Sung et al., 2009). Sung and colleagues found that working memory capacity effects emerged most prominently under the more syntactically challenging conditions from the Revised Token Test when the stimuli are only temporarily available. However, the token tests carry some caveats in that action verbs have a limitation for assigning the thematic roles to the noun phrases using tokens given that the tokens are not the best agents and themes to represent various actions.

In order to overcome this limitation, but theoretically motivated by working memory demands on the Token-based tests, we developed a novel syntactic assessment battery by employing humanized color pictograms that make the syntactic stimuli fully semantically reversible. Given that these color pictograms are humanized, various action verbs can be combined with the noun phrases. We also manipulated the levels of syntactic complexity including three different sentence types such as (1) active sentences with 2-place verbs, (2) active sentences with 3-place verbs, and (3) passive sentences by varying the word order. The syntactic complexity has been reported as one of the critical factors that need to be considered when developing a theoretically motivated linguistic measure (Thompson et al., 2003). We specifically focused on the syntactic complexity from the perspectives of the syntactic computations (e.g., active vs. passive comparisons) and sentence length (e.g., active with 2-place vs. 3-place verbs) Most recently, we reported the clinical utility for differential diagnosis of mild cognitive impairment (MCI) (Sung et al., 2020). They reported that the syntactic computational loads served as a critical linguistic marker to differentiate people with MCI from normal aging, when the fully semantically reversible structures were used by minimizing the top-down reliance. More studies are in progress to examine the systematic effects of complexity that may differentially affect various types of neurogenic communication disorders.

We reported the preliminary results of this novel sentence comprehension test in a series of articles that examined the effects of aging, word order, and working memory (Sung, 2015a,b; Sung et al., 2017). Among various factors, we specifically focused on age and education effects on the novel test, given that these are among critical factors that serve as a cognitive reserve (Stern, 2002) when people perform linguistic and cognitive tasks. The cognitive reserve hypothesis suggested that people with higher education are less vulnerable to aging, given that education serves as underlying cognitive mechanisms that are responsible for flexible reallocations of limited cognitive resources. For extending its clinical usage, we aim to examine the age and education effects of the test and provide descriptive data stratified by age groups and educational levels.

## Methods

### Participants

A total of 232 elderly adults (age > 60) who were monolingual Korean speakers consented to participate in the study, which was approved by the Institutional Review Board of Ewha Womans University (No. 116-1). We administered a health-screening questionnaire (Christensen et al., 1991) and the Korean Version of the Mini-Mental State Examination (K-MMSE; Kang, 2006). Thirty-seven participants were excluded because they did not meet the normal inclusion criteria, resulting in a total of 195 elderly adults in the final data analyses. All participants exhibited age- and education-adjusted normal range on the K-MMSE (>16 %ile). The mean age was 71.59 years (*SD* = 8.16, Range = 60–90), and the mean years of education was 10.03 (*SD* = 4.94, Range = 0–20).

### Construction of the Syntactic Assessment Battery

**FIGURE 1:**
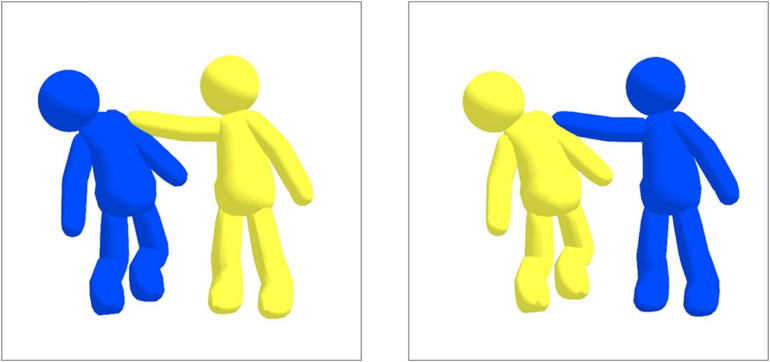
An example sentence and picture from the Syntax Assessment Battery. Target sentence **(Left)**: The Yellow pushes the Blue. Syntactic foil sentence **(Right)**: The Blue pushes the Yellow.

The novel Syntactic Assessment Battery (SAB) is based on a sentence-picture matching paradigm, in which all sentences are semantically reversible. In order to minimize the influence of top-down semantic processing on syntactic comprehension, humanized pictograms were employed with three colors (yellow, black, blue) that denote each thematic role in a sentence. For color selection, we adjusted the five colors (black, blue, green, red, and white) used in the Revised Token Test (RTT; McNeil and Prescott, 1978) to three colors by reflecting the linguistical and cultural factors of Korean. Since “blue” and “green” are used interchangeably among elderly adults in Korea (Koo, 2008) and, furthermore, these two colors are very close in color frequency of the visual spectrum (Bruno and Svoronos, 2005), we only included “blue,” which has a higher lexical frequency than “green.” The color “red” was excluded due to cultural and ideologically related issues. During the Korean War, the “red” color represents the army of North Korea. For older generations who have gone through the Korean War, the “red” color represents an ideologically sensitive notion. For these reasons, we eliminated the color “red.” Finally, we replaced the color “white” with “yellow” to increase the visibility of the pictograms in the SAB. All human pictograms were produced in the same form, and the colors and actions of the characters changed according to each thematic role in a target sentence. Figure 1 presents an example of SAB.

The Korean language allows the freedom to scramble word order as far as verbs are placed at the end of a sentence. The canonical word order in Korean follows “Subject + Object + Verb” (SOV), but the non-canonical word order (OSV) structure is also possible. We manipulated the canonicity of word order in the SAB by including both SOV and OSV structures. Korean has a case marking system as a postposition to denote the structural functions. For example, when the nominative case marker (ka) is attached to the noun, the noun phrase (NP) with ka-case marker represents a subject in a sentence, and NP with an accusative case marker (lul) represents an object.

The SAB consisted of three sentence types: (1) active sentences with 2-place verb (A2) (e.g., The Yellow pushes the Blue), active sentences with 3-place verb (A3) (e.g., The Blue gives a box to the Black), and a passive sentence (P) (e.g., The Blue is pushed by the Yellow). In the process of Korean passivization, the theme is moved to the subject position marked by the nominative case, and an agent is represented by the oblique case marker (eykey), which is equivalent to *by-phrase* in English.

Each structure is presented in two different word orders of canonical (C) and non-canonical (NC) structures, resulting in a total of 6 different syntactic structures (A2-C, A2-NC, A3-C, A3-NC, P-C, and P-NC). Each syntactic structure consisted of six items, leading to a total of 36 items. We provided example sentences for each syntactic structure using the Yale Romanization transliteration system (Martin, 1992) as follows.


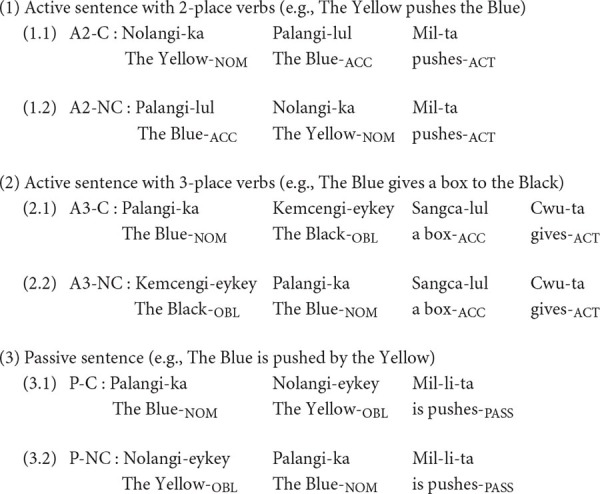


### Experimental Procedures and Scoring System

Participants were asked to point to the picture which corresponds to the sentence, which was presented auditorily. For each item, a target (e.g., The Yellow pushes the Blue) and a syntactic foil (e.g., The Blue pushes the Yellow) pictures were displayed. We pseudo-randomized sentence stimuli so that no more than 3 items with the same sentence type were presented consecutively.

Prior to the administration of the main test, participants were asked to complete four practice trials to verify that their color perceptions of the stimuli were accurate. When participants asked examiners to repeat the sentence, only one repetition of the stimulus was allowed and the final response was recorded. Each item was scored as 1 for correct and 0 for incorrect, resulting in a total of 36 scores.

### Statistical Analyses

We applied a multiple linear regression model using R (version 3.6.0) to explain age and education effects. Among model candidates, the optimal model was chosen by using the Akaike information criterion (AIC), a widely used criterion for comparing models to maintain predictive power in statistical analyses.

## Results

### Demographic Characteristics and Grouping Rationales for Age and Educational Levels

**FIGURE 2:**
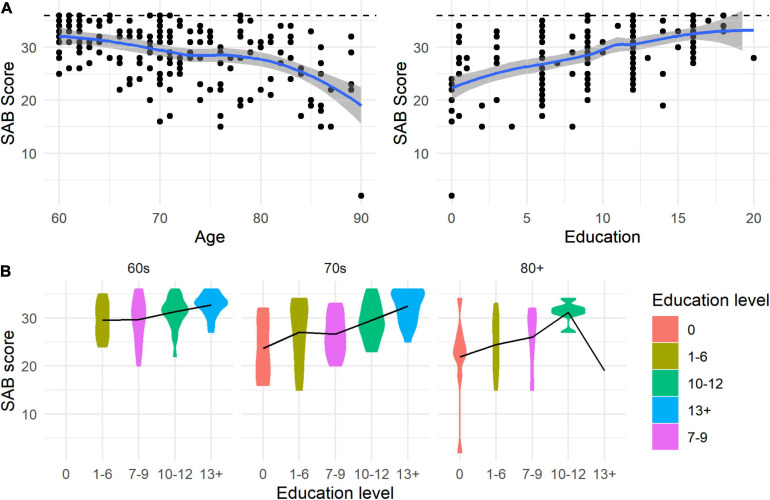
Locally estimated scatterplot smoothing (LOESS) result **(A)** and distribution of SAB for each age and education level **(B)**.

Prior to the application of linear regression models to display regression-based norms from the current dataset, we examined whether linear relations among the variables could be assumed. Based on visual inspection of Figure 2A, we discovered nonlinear effects of age and education on the response variables. In order to handle nonlinearity, we categorized age and education into three (60–69, 70–79, and 80–90) and five (0 = no formal education, 1–6 = elementary school graduates, 7–9 = middle school graduates, 10–12 = high school graduates, and >13 = college education and above) groups. Table 1 presents means and standard deviations for each subtest with the overall scores in each age and education group, when the sample size for each group is greater than 10. The threshold on the sample size is suggested to maintain the balance between the accuracy of two sample statistics, mean and variance, and the interpretability of the data set. This result was obtained by a simulation study that generates 1,000 standard normal samples of each size (3–30 observations) and computes the estimated error of sample statistics. The distribution of total scores for each age and education group was visualized using a violin plot in Figure 2B.

**TABLE 1:** Descriptive information for each age and education group.

		**Total**	**Subtest 1 A2-C**	**Subtest 2 A2-NC**	**Subtest 3 A3-C**	**Subtest 4 A3-NC**	**Subtest 5 P-C**	**Subtest 6 P-NC**
		
	**Education level**	**Mean (SD)**	**Mean (SD)**	**Mean (SD)**	**Mean (SD)**	**Mean (SD)**	**Mean (SD)**	**Mean (SD)**
Age—60s	Edu 7–9 (*n* = 17)	29.6471 (4.6360)	5.76 (0.437)	4.41 (1.87)	5.65 (0.61)	4.00 (1.27)	5.18 (1.19)	4.47 (1.66)
	Edu 10–12 (*n* = 29)	31.2759 (3.1041)	5.97 (0.186)	5.14 (1.06)	5.48 (0.69)	4.62 (0.98)	5.52 (0.63)	4.79 (1.05)
	Edu 13+ (*n* = 27)	32.7407 (2.3303)	5.85 (0.362)	5.22 (1.05)	5.89 (0.32)	4.93 (0.96)	5.85 (0.36)	5.11 (1.09)
Age—70s	Edu 1–6 (*n* = 17)	27.0588 (6.3391)	5.35 (0.86)	3.82 (1.70)	5.00 (1.32)	3.59 (1.42)	4.76 (1.35)	4.47 (1.18)
	Edu 7–9 (*n* = 13)	26.6154 (4.2336)	5.62 (0.65)	4.00 (2.00)	5.54 (0.66)	2.92 (1.66)	5.15 (0.89)	3.38 (1.71)
	Edu 10–12 (*n* = 19)	29.5263 (3.8495)	5.79 (0.54)	4.53 (0.98)	5.68 (0.58)	3.95 (1.35)	5.26 (1.19)	4.32 (1.29)
	Edu 13+ (*n* = 22)	32.5000 (3.3345)	5.77 (0.43)	5.27 (1.16)	5.73 (0.70)	4.86 (1.42)	5.64 (0.73)	5.23 (1.11)
Age—80s	Edu 1–6 (*n* = 15)	24.4667 (5.4885)	5.20 (1.21)	3.60 (2.06)	4.73 (0.88)	3.73 (1.79)	3.73 (1.39)	3.47 (1.13)

**A2-C, active sentence with 2-place verbs in a canonical word order; A2-NC, active sentence with 2-place verbs in a non-canonical word order; A3-C, active sentence with 3-place verbs in a Canonical word order; A3-NC, active sentence with 3-place verbs in a non-canonical word order; P-C, passive sentence in a canonical word order; P-NC, passive sentence in a non-canonical word order; SD, standard deviation; Edu, education level.**

### Regression-Based Analyses of Age and Education Effects on the Test

We applied a multiple linear regression model to examine the age and education effects on the SAB. Dummy coding was used for each factor variable. The baseline for age was 60–69 years (as the youngest group). For education, the baseline was set at 1–6 years, which was the mandatory educational level supported by the government for that generation in South Korea.

Grouping a continuous variable usually incurs a trade-off between exploration and exploitation because having a larger number of groups loses less information but increases model complexity. Due to the nature of linear regression models, we expect that the average test scores of all possible pairs will be statistically different. This notion can be simultaneously tested by performing Tukey’s test with a significance level *a* for all. Although the test is conservative for unbalanced data, most of the pairs for each age and education variable had statistically different means with adjusted *p*-value less than 0.05 for the significance level *a*0.05.

We fitted six candidate models up to first-order interactions: (1) age; (2) education; (3) age and education; and (4) age, education, and their interaction term. The corresponding AIC values for the models were (1) 630.84, (2) 607.36, (3) 601.15, and (4) 602.43; based on these, we chose the age and education model (3) as the final model, considering that a lower AIC value indicates a better fit (Bozdogan, 1987). For the selected model, Table 2 presents the estimated coefficients and *p*-values for each outcome variable including total scores and six subtest scores.

**TABLE 2:** Statistical results from the multiple linear regression models.

**Model**	**Total**	**Subtest 1**	**Subtest 2**	**Subtest 3**	**Subtest 4**	**Subtest 5**	**Subtest 6**
**(3) Age and education**		**A2-C**	**A2-NC**	**A3-C**	**A3-NC**	**P-C**	**P-NC**
Intercept	EC = 28.5025 W = 30.249 *p* < 0.001***	EC = 5.5896 W = 39.789 *p* < 0.001***	EC = 3.9945 W = 12.606 *p* < 0.001***	EC = 5.1942 W = 29.587 *p* < 0.001***	EC = 4.3874 W = 15.688 *p* < 0.001***	EC = 4.9723 W = 22.482 *p* < 0.001***	EC = 4.4012 W = 16.398 *p* < 0.001***
Age—70s	EC = −1.5267 W = −2.041 *p* = 0.0427*	EC = −0.1491 W = −1.337 *p* = 0.1830	EC = −0.2418 W = −0.961 *p* = 0.3376	EC = −0.1063 W = −0.763 *p* = 0.4466	EC = −0.6449 W = −2.905 *p* = 0.0041**	EC = −0.2375 W = −1.352 *p* = 0.1778	EC = −0.2298 W = −1.078 *p* = 0.2822
Age—80s	EC = −3.4099 W = −3.268 *p* = 0.0013**	EC = −0.4035 W = −2.594 *p* = 0.0102 *	EC = −0.5112 W = −1.457 *p* = 0.1468	EC = −0.3974 W = −2.044 *p* = 0.0423*	EC = −0.6355 W = −2.052 *p* = 0.0415*	EC = −0.9236 W = −3.772 *p* < 0.001***	EC = −0.6093 W = −2.050 *p* = 0.0417*
No formal Edu	EC = −3.2291 W = −2.359 *p* = 0.0194*	EC = −0.7975 W = −3.908 *p* < 0.001***	EC = −0.2886 W = −0.627 *p* = 0.5314	EC = −0.5492 W = −2.153 *p* = 0.0326*	EC = −0.4978 W = −1.225 *p* = 0.2220	EC = −0.7864 W = −2.447 *p* = 0.0153*	EC = −0.3329 W = −0.854 *p* = 0.3943
Edu 7–9	EC = 0.5516 W = 0.505 *p* = 0.6139	EC = 0.1805 W = 1.109 *p* = 0.2688	EC = 0.3398 W = 0.926 *p* = 0.3558	EC = 0.3878 W = 1.907 *p* = 0.0581	EC = −0.5999 W = −1.852 *p* = 0.066	EC = 0.3050 W = 1.190 *p* = 0.2354	EC = −0.1430 W = −0.460 *p* = 0.6460
Edu 10–12	EC = 3.0617 W = 3.055 *p* = 0.0026**	EC = 0.3780 W = 2.530 *p* = 0.0122*	EC = 1.0363 W = 3.075 *p* = 0.0024**	EC = 0.4429 W = 2.372 *p* = 0.0187*	EC = 0.2805 W = 0.943 *p* = 0.3469	EC = 0.6583 W = 2.799 *p* = 0.0057**	EC = 0.3955 W = 1.386 *p* = 0.1674
Edu 13+	EC = 4.5974 W = 4.431 *p* < 0.001***	EC = 0.3040 W = 1.965 *p* = 0.0509	EC = 1.2822 W = 3.675 *p* < 0.001***	EC = 0.6605 W = 3.417 *p* < 0.001***	EC = 0.7291 W = 2.367 *p* = 0.0189*	EC = 0.8507 W = 3.493 *p* < 0.001 ***	EC = 0.8321 W = 2.815 *p* = 0.0054**

A2-C, active sentence with 2-place verbs in a canonical word order; A2-NC, active sentence with 2-place verbs in a non-canonical word order; A3-C, active sentence with 3-place verbs in a canonical word order; A3-NC, active sentence with 3-place verbs in a non-canonical word order; P-C, passive sentence in a Canonical word order; P-NC, passive sentence in a non-canonical word order; EC, estimated coefficient; W, Wald’s statistics; p, p-value (*0.05/**0.01/***0.001).

## Discussion

The current study investigated age and education effects on a novel syntactic assessment battery, which was developed for early detection of aging-related decline in sentence processing abilities. Several versions of sentence comprehension tests have used a variety of assessment paradigms such as sentence-picture matching (Caplan et al., 1997; Small et al., 1997), plausibility judgment (Rochon et al., 1994; Meyer et al., 2012), grammaticality judgment tasks (Kempler et al., 1998; Franck et al., 2015), and so on. However, relatively few studies have reported normative data for syntactic assessment tests which are specifically targeted to elderly populations.

The present study developed a novel syntactic assessment battery using a sentence-picture paradigm. The test is novel in the sense that we limited top-down semantic influence on performance when testing the abilities to parse syntactic units with a greater variety of action verbs than previous tests (e.g., Token Test). Furthermore, the current test has been validated through a series of the preliminary studies by Sung and colleagues by comparing young and old groups (Sung, 2015a,b; Sung et al., 2017). A most recent research on the SAB suggested that the syntactic complexity serves as a critical linguistic marker for differential diagnosis of mild cognitive impairment (MCI) from normal aging populations (Sung et al., 2020). The authors reported that individuals with MCI did not significantly differ from normal elderly adults in a simple active syntactic form, whereas they performed significantly worse on the passive sentences, which are linguistically more complex and challenging to process than active sentences. The passive sentences required additional linguistic and cognitive processes to go through in order to map its surface structure with the NP movement to the deep structure. As delineated in the methods on the passivization process of Korean, the NP movement occurs from its original place as in the object position to the subject position in its surface structure, although the thematic role of the moved NP represents a theme, which is not canonically placed in a subject position. Sung et al. (2020) confirmed that individuals with MCI behaviorally demonstrated greater difficulties in the syntactic structures that are claimed to be theoretically more complex. Furthermore, the authors tested the models to elicit the most efficient combination of the subtests among three models: (1) only active sentences, (2) only passive sentences, and (3) both active and passive sentences. Interestingly, they found that the complex sentence condition with only passive sentences was good enough to be administered to generate equivalent performance on the condition in which both sentence types are employed. The results suggested the potential extension of its clinical utility and efficiency for early detection of aging effects and early dementia.

To advance its clinical use for elderly adults, we attempted to provide a wide range of data on age and education effects for elderly populations including all subtests and total scores in the present study. The current results for the total scores suggest that the syntactic assessment task effectively detected age and education effects on sentence processing for elderly adults, indicating that people generally demonstrated worse performance as age increases with age, but better performance as educational levels increased. The effects were confirmed by the statistical results of significant age and education effects in the regression model. It should be noted that the effect of age was nonlinear, suggesting that participants in their 80s demonstrated greater difficulties than participants in their 70s and 60s. Regarding the age effects for each subtest, we observed that age effects were most prominent for the 80s age group compared to the baseline 60s group across all subtests. In contrast, the 70s age group performed worse than the 60s age group only for Subtest 4 (A3-NC). Subtest 4 contains the longest, active sentences with 3-place verbs and non-canonical word order. It is important to note that sentence length and word order affected the 70s age group but not the baseline 60s group, whereas the remaining subtests did not differentiate the 70s and 60s age groups. Subtest 4 has additional argument structures, with three arguments using ditransitive verbs compared to subtest 1 and 2, which are active sentences with two argument structures using the transitive verbs. The current results suggested that the 70s age group was differentially influenced by the number of arguments structures that are associated with the sentence length. Several researchers claimed that the number of argument structures is one of the critical factors that affect sentence processing, especially when people have a disrupted nervous system due to stroke. A series of studies reported that people with aphasia after stroke demonstrated systematically greater difficulties as the number of argument structures increases (Shapiro et al., 1993; Thompson et al., 1995). Unlike English, Korean syntactic structures are allowed to be scrambled. Considering that the subtest 4 carries non-canonical word order (OSV), the 70s age group presented greater burden on parsing sentences when the word order was not canonical especially with increased numbers of argument structures. The results suggested that it is critical to consider both argument structures and word order to detect aging effects.

With respect to education effects, the group with no formal education demonstrated significantly worse performance than the baseline group (1–6), whereas the 10–12, and 13+ groups presented better performance than the baseline group. The lowest education group (no formal education) scored on average 3.2 points less on total scores compared to the baseline group. On the other hand, people who attended high school (10–12) or more (Edu 13+) attained at least 3 more points in the test. It is interesting that the 7–9 group was not significantly different from the baseline group across the board. The baseline group (elementary school) reflects the mandatory education system for the older generation in South Korea, whereas the mandatory education is up to 9 years (middle school) in recent years for the younger generation. The current results suggest that the educational levels between elementary and middle school did not differ in this novel syntactic assessment task for elderly adults. In contrast, people with higher education generally demonstrated stronger performance on the test. The current results are in line with the previous studies that suggested that education serves as a cognitive reserve (Stern, 2002, 2009). The cognitive reserve hypothesis by Stern (2002) posits that cognitive capacity subserves underlying mechanisms that are flexibly and efficiently allocated to handle task demands by employing the optimal cognitive strategies. Stern suggested that this concept accounts for better performance on behavioral testing results for individuals with higher levels of intelligence and education and explains why education turns out to be a significant predictor that is less vulnerable to aging-related cognitive decline.

The current study suggests that this novel syntactic assessment battery reflected age and education effects satisfactorily, indicating that this test can be used to sensitively detect those effects. Early detection of aging effects is important especially in countries with a rapidly increasing ratio of older generations. Considering that many countries have already entered into an aged society with many socioeconomic burdens related to the elderly, the systematic and reliable monitoring of linguistic and cognitive decline in older persons is critical for providing better clinical service to prevent dementia. The current work contributes to providing effective linguistic screening procedures for early detection of aging effects with education taken into account. As further studies, we are planning to report a series of updated normative data for the SAB every 3–5 years to modify and refine the current work for its wide usage as a clinical tool to differentiate aging effects from early symptoms of dementia.

## Data Availability Statement

The original contributions presented in the study are included in the article/Supplementary Material, further inquiries can be directed to the corresponding author/s.

## Ethics Statement

The studies involving human participants were reviewed and approved by the Institutional Review Board of Ewha Womans University. The patients/participants provided their written informed consent to participate in this study.

## Author Contributions

JS contributed to conception and design of the study and wrote the first draft of the manuscript. JS and SC organized the database. HA performed the statistical analysis and interpretation under supervision of JS and KL. HA and SC wrote sections of the manuscript. All authors contributed to manuscript revision, read, and approved the submitted version.

## Conflict of Interest

The authors declare that the research was conducted in the absence of any commercial or financial relationships that could be construed as a potential conflict of interest.
